# Three-Dimensional Anthropometric Analysis of the Effect of Lip Reconstructive Surgery on Children with Cleft Lip and Palate at Three Different Times †

**DOI:** 10.3390/children11070824

**Published:** 2024-07-05

**Authors:** Gabriela Mendonça Rando, Eloá Cristina Passucci Ambrosio, Paula Karine Jorge, Chiarella Sforza, Márcio Menezes, Ana Lúcia Pompeia Fraga de Almeida, Simone Soares, Gisele Silva Dalben, Cristiano Tonello, Cleide Felício Carvalho Carrara, Maria Aparecida Andrade Moreira Machado, Thais Marchini Oliveira

**Affiliations:** 1 Department of Pediatric Dentistry, Orthodontics and Public Health, Bauru School of Dentistry, University of São Paulo, Bauru 17012-901, Brazil; 2 Hospital for Rehabilitation of Craniofacial Anomalies, University of São Paulo, Bauru 17012-900, Brazil; 3 Department of Biomedical Sciences for Health, Functional Anatomy Research Center, Faculty of Medicine and Surgery, University of Milan, 20133 Milano, Italy; 4 School of Health Science, State University of Amazonas, Manaus 69065-001, Brazil; 5 Department of Prosthodontics and Periodontology, Bauru School of Dentistry, University of São Paulo, Bauru 17012-901, Brazil; 6 Bauru School of Medicine, University of São Paulo, Bauru 17012-901, Brazil

**Keywords:** cleft lip, cleft palate, dental arch, growth and development, imaging, three-dimensional

## Abstract

Objectives: This investigation aimed to assess the optimal timing for lip repair in children with cleft lip and palate via 3D anthropometric analysis to evaluate their maxillofacial structures. Methods: The sample comprised 252 digitized dental models, divided into groups according to the following timing of lip repair: G1 (n = 50): 3 months; G2 (n = 50): 5 and 6 months; G3 (n = 26): 8 and 10 months. Models were evaluated at two-time points: T1: before lip repair; T2: at 5 years of age. Linear measurements, area, and Atack index were analyzed. Results: At T1, the intergroup analysis revealed that G1 had statistically significant lower means of I-C′, I-C, C-C′, and the sum of the segment areas compared to G2 (*p* = 0.0140, *p* = 0.0082, *p* = 0.0004, *p* < 0.0001, respectively). In addition, there was a statistically significant difference when comparing the cleft area between G2 and G3 (*p* = 0.0346). At T2, the intergroup analysis revealed that G1 presented a statistically significant mean I-C′ compared to G3 (*p* = 0.0461). In the I-CC’ length analysis, G1 and G3 showed higher means when compared to G2 (*p* = 0.0039). The I-T′ measurement was statistically higher in G1 than in G2 (*p* = 0.0251). In the intergroup growth rate analysis, G1 and G2 showed statistically significant differences in the I-C′ measurement compared to G3 (*p* = 0.0003). In the analysis of the Atack index, there was a statistically significant difference between G1 and the other sample sets (*p* < 0.0001). Conclusion: Children who underwent surgery later showed better results in terms of the growth and development of the dental arches.

## 1. Introduction

Cleft lip and/or palate represent the most frequent malformations that affect the human species and are easily diagnosed even in prenatal life, which is why they are so widely studied [1]. They manifest in the embryonic period of intrauterine life and have a multifactorial etiology, associating genetic and environmental factors. The process of morphological rehabilitation of clefts begins with primary plastic surgery on the lip (lip repair) at three months of age and plastic surgery on the palate (palatoplasty) around one year of age [1]. However, the rehabilitation process extends beyond the anatomical repair of the cleft. Depending on the type and extent of the cleft, several other functional and morphological impairments such as speech, hearing, occlusion development, and craniofacial growth accompany the individual with clefts, requiring the intervention of the interdisciplinary team at opportune times to achieve the comprehensive rehabilitation of the patient with this malformation [1,2].

Primary surgeries have a paradoxical effect on individuals with cleft lip and palate because they have a marked and progressive restriction of anteroposterior maxillary growth [2]. The tension in the reconstructed lip and the scar caused by lip repair restricts the anterior development of the maxilla. Early palatoplasty also seems to have a restrictive influence, albeit to a lesser degree compared to lip repair, on sagittal maxillary growth [3,4]. This restrictive effect on maxillary growth eventually causes a Class III skeletal pattern due to maxillary deficiency [5]. The present study is justified due to the absence of longitudinal studies that have digitally evaluated the dental arches of children with unilateral cleft lip and palate before and after three different times of lip reconstructive surgery. Thus, the purpose of this study was to determine which is the best surgical time to perform lip repair surgery, assessing the effect on the jaws of children with cleft lip and palate via 3D analysis at three different times.

## 2. Materials and Methods

### 2.1. Sample Selection

The present retrospective study was approved by the Institutional Review Board (CAAE: 46104721.2.0000.5417). Plaster dental models are part of the institutional rehabilitation protocol; thus, a consent form was not necessary. The sample inclusion criteria were children with unilateral cleft lip and palate, of both genders, operated by the same surgical technique, attending the rehabilitation center without prior surgery, and who presented complete documentation before lip repair and from 5 years of age. The exclusion criteria were the presence of syndromes or associated malformations and children with incomplete documentation.

The sample size was calculated (paired *t*-test) so that the selected number of children represented the minimum number for the study to be conducted. For this, the study of Ambrosio et al. (2022) [6] was considered with a standard deviation of 3.88 mm of anteroposterior dental arch length (I-TT’). The calculation considered a significance level of 5%, test power of 80%, and a minimum difference to be clinically detected of 3.50 mm. The minimum size of each sample group was 20 children.

Children were divided into three groups according to the time of surgical intervention of each participant obtained from their medical records: Group 1 (G1)—50 children operated at 3 months of age via the Millard technique for lip closure and total palate repair at 12 months via the von Langenbeck technique; Group 2 (G2)—50 children operated between 5 and 6 months of age via the Millard technique for lip repair and total palate repair at 12 months via the von Langenbeck technique; and Group 3 (G3)—26 children operated between 8 and 10 months of age via the Millard technique for lip repair and total palate repair at 12 months by the von Langenbeck technique. The children’s legal guardians received guidance regarding surgical care from doctors and nurses. Cues include cleaning the area with saline solution or boiled water. In addition, legal guardians were advised regarding signs of infection in the surgical wound. The cutaneous suture was removed on the 7th postoperative day (in the city of origin or in the institution where the procedure was performed). The physiotherapist also guided the need to perform postoperative lip massage to reduce edema and pain and to improve blood circulation in the operated area and, consequently, healing.

The evaluation was performed on three-dimensional digital models of each participant. A previously trained and calibrated examiner performed the assessment. The models were evaluated at Time 1 (T1): before lip repair surgery; and at Time 2 (T2): after 5 years of age.

### 2.2. Obtaining the Models

The plaster models were obtained from the documentation files of the hospital. The models underwent a scanning process via a 3D scanner (3Shape’s R700^TM^ Scanner, 3Shape AS, Copenhagen, Denmark) coupled to a computer. The models were accurately scanned, and the measurements were obtained by the stereophotogrammetry system software (Mirror imaging software, version 2.8.3, Canfield Scientific, Inc., Fairfield, NJ, USA) [6].

### 2.3. Linear Measurements

**Figure 1:**
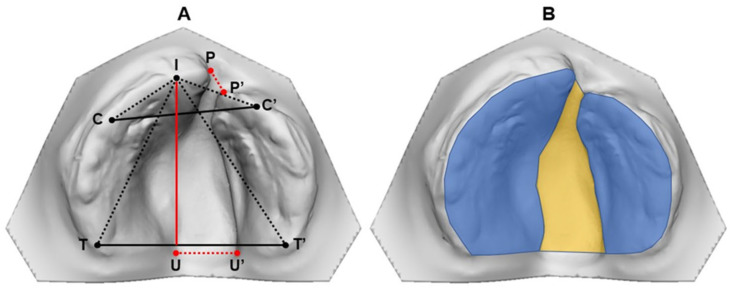
Anthropometries evaluated on the dental models of the upper dental arches in participants with unilateral cleft lip and palate. (**A**) Linear measurements: intercanine (C-C′) and intertuberosity (T-T′) distances; anterior (I-CC′) and anteroposterior dental arch lengths (I-TT′); anterior dental arch distances on the side without cleft (I-C) and with cleft (I-C′); anteroposterior dental arch distances on the side without cleft (I-T) and with cleft (I-T′); anterior (P-P′) and posterior (U-U′) cleft amplitudes. (**B**) Delimitations of the palatal bone segments (blue color) and the cleft (yellow color) for analyzing the area of the dental arches.

The measurements were performed directly on the three-dimensional models. On the images, the demarcation of points and lines, as well as the area of upper dental arches, was performed according to previous study [6]. The following linear measurements (Figure 1) in mm were evaluated:

- Intercanine distance (C-C′): Determined by points C and C′, the distance between the right and left lateral sulci of the alveolar ridge crest.
- Intertuberosity distance (T-T′): Determined by points T and T′, a transverse line joining the end of the alveolar ridge on the right and left sides.
- Anterior dental arch length (I-CC′): Determined by the straight line from the interincisor point (I) passing perpendicularly to the intercanine distance line (C-C′).
- Anteroposterior dental arch length (I-TT′): Determined by the straight line from the interincisor point (I) that passes perpendicularly to the intertuberosity distance line (I-TT′).
- Anterior dental arch distance on the side without cleft (I-C): Determined by the interincisor points (I) and point C (cusps of the deciduous canine on the greater bone).
- Anterior distance of the dental arch on the cleft side (I-C′): Determined by interincisor points (I) and point C′ (cusps of deciduous canine in the small bone).
- Anteroposterior distance of the dental arch on the cleft side (I-T′): Determined by interincisor points (I) and point T′ (intertuberosity—bulging at the end of the alveolar ridge of the minor bone).
- Anteroposterior distance of the dental arch on the side without cleft (I-T): Determined by interincisor points (I) and point T (intertuberosity—bulging at the end of the alveolar ridge of the greater bone).
- Anterior cleft width (P-P′): Determined by points P (beginning of the anterior alveolar ridge of the major bone segment) and point P′ (beginning of the anterior alveolar ridge of the minor bone segment).
- Posterior cleft width (U-U′): Determined by points U (posterior point on the fissure edge of the major bone line) and U′ (posterior point on the fissure edge of the minor bone line).

### 2.4. Dental Arch Area

Before lip repair (Time 1), the area of the major and minor segments was delimited by points passing through the crest of the alveolar ridge and contouring the palatal process, with the limit at the most posterior end of the tubercle [6]. For intra- and intergroup comparative analysis, the areas of palatal segments were summed. The cleft area was delimited anteriorly and posteriorly by a line joining the points at the ends of the alveolar ridges between the major and minor segments (Figure 1). From 5 years of age (Time 2), in the presence of teeth, the points for delimiting the area passed through the palatal gingival margin of teeth with the posterior limit at the T-T′ distance. The area was evaluated in mm^2^.

### 2.5. Atack Index

The three-dimensional images of the models were evaluated by applying the index of Atack et al. (1997) [7]. This index defines the systematization criteria by which to qualify and quantify the occlusion morphology in individuals with unilateral cleft lip and palate, comprising a scale from 1 to 5 with an increasing degree of severity and considering the interarch relationship, the upper dental arch shape, and the inclination of upper incisors.

### 2.6. Statistical Analysis

GraphPad software Version 5.0 (Prism 5 for Windows., Inc., San Diego, CA, USA) was used in data analysis with a significance level of 5%. The inter-examiner analysis of the methodology was performed via the re-measurement of 1/3 of the sample after an interval of two weeks. The intraclass correlation coefficient was used to assess reliability. The Shapiro–Wilk test was applied to determine normality. Paired *t*-test and Wilcoxon test were applied in intragroup analyses. ANOVA followed by Tukey’s Test and Kruskal–Wallis test followed by Dunn’s test were used in the intergroup analyses. Descriptive data were presented as mean/standard deviation, median/interquartile range, grouping (number of participants), and percentage.

## 3. Results

Group 1 included 50 children (17 girls and 33 boys), Group 2 included a further 50 children (20 girls and 30 boys), and Group 3 comprised 26 children (13 girls and 13 boys), totaling 126 children (50 girls and 76 boys) and 252 dental models. Regarding age, in Group 1, participants were 0.27 (±0.02) years old at Time 1 and 6.49 (±0.79) years old at Time 2; in Group 2, participants were 0.46 (±0.06) years old at Time 1 and 5.98 (±0.58) years old at Time 2; and in Group 3, participants were 0.67 (±0.18) years old at Time 1 and 6.16 (±0.80) years old at Time 2. The high correlation between measurements performed by the examiner indicated that the measurements were sufficiently reproducible (r = 0.957, Intraclass Correlation Coefficient).

In Group 1, there was a statistically significant difference in the measurements I-C′, C-C′, I-CC′, and the sum of areas with lower values in T2 (linear measurements, *p* < 0.0001, and sum of the segment areas, *p* = 0.003); while I-C, I-T′, I-T, T-T′, and I-TT′ were significantly larger values at Time 2 (*p* < 0.0001) (Table 1). In Group 2, there was a statistical difference in the measurements I-C′, C-C′, I-CC′, and the sum of the segment areas with lower values in T2 (*p* < 0.0001, *p* < 0.0001, *p* < 0.0001, *p* = 0.0466, respectively); in contrast, I-C, I-T′, I-T, T-T′, and I-TT′ were significantly larger values at Time 2 (*p* = 0.0056, *p* = 0.0084, *p* < 0.0001, *p* < 0.0001, and *p* = 0.0005, respectively) (Table 1). In Group 3, there was a statistically significant difference in measurements I-C′, C-C′, I-CC′, and the sum of the segment areas with lower means in T2 (*p* < 0.001, *p* = 0.0007, *p* = 0.0009, and *p* = 0.0170, respectively), while I-T′, I-T, and T-T′ were significantly larger values at Time 2 (*p* = 0.0083, *p* < 0.0001, *p* < 0.0001, respectively) (Table 1).

At Time 1, the intergroup analysis revealed that Group 1 had statistically significant lower means of I-C′, I-C, C-C′, and the sum of the segment areas compared to Group 2 (*p* = 0.0140, *p* = 0.0082, *p* = 0.0004, *p* < 0.0001, respectively). In addition, there was a statistically significant difference when comparing the cleft area between Group 2 and Group 3, in which Group 2 had the lowest mean (*p* = 0.0346) (Table 2).

At Time 2, the intergroup analysis revealed that Group 1 presented a statistically significant mean I-C′ compared to Group 3 (*p* = 0.0461). In the I-CC′ length analysis, Groups 1 and 3 showed higher means when compared to Group 2 (*p* = 0.0039). The I-T′ measurement was statistically higher in Group 1 than in Group 2 (*p* = 0.0251). There was a statistically significant difference when comparing I-T, T-T′, and the sum of the segment areas between Group 1 × Group 2 × Group 3, where Groups 2 and 3 had the highest values (*p* = 0.0030, *p* < 0.0001, *p* < 0.0001, respectively) (Table 3).

In the intergroup growth rate analysis, Groups 1 and 2 showed statistically significant differences in the I-C′ measurement compared to Group 3 (*p* = 0.0003). There was a statistically significant difference in comparing the I-C measurement between Group 1 and Group 2 (*p* = 0.0001). There was also a statistically significant difference when comparing measurements C-C′ and I-CC′, in which Group 2 presented lower values (*p* = 0.0002 and *p* = 0.0001, respectively). Measurement I-T showed a higher median in Group 1 than in the other sample sets (*p* < 0.0001). In the I-TT’ length, there was a statistically significant difference between Group 1 and Group 3, in which the lowest value was observed in Group 3 (*p* = 0.0071) (Table 4).

In the analysis of the Atack index, Group 1 had the largest number of participants with index 4 (n = 22, 44%), while in Groups 2 and 3, index 3 was the most frequent (Group 2, n = 24, 48%; Group 3, n = 11, 42.4%). There was a statistically significant difference between Group 1 and the other sample sets (*p* < 0.0001) (Table 5).

## 4. Discussion

The surgical repair of cleft lip and palate occurs in the first year of life [8,9,10,11,12,13,14,15,16,17,18,19,20]; after these surgeries, these individuals have pronounced skeletal discrepancies in anteroposterior, transverse, and vertical directions, with a Class III skeletal pattern. This occurs due to the formation of fibrous tissue in the cleft area, which impairs maxillary growth. The literature is still quite conflicted regarding the surgical time of lip repair and palatoplasty [21,22,23,24]. Aiming to minimize this effect, this study aimed to compare the ages at which lip repair was performed with the maxillary growth of these children from the age of five. It is essential to highlight that the institutional protocol indicates that lip surgical repair should ideally be performed at three months of age [2]. Thus, the evaluation of participants operated on late occurred due to adverse reasons inherent to the patients themselves, such as absenteeism or the child not having the clinical and/or laboratory conditions to undergo the surgical procedure.

In this study, Group 1, Group 2, and Group 3 showed some smaller dental arch measurements at Time 2 when compared to Time 1; i.e., the primary surgeries negatively affected the dental arch growth, with a reduction of the anterior maxilla. However, Groups 2 and 3 had higher means (I-C′; C-C′; I-CC′, and Area) than Group 1; i.e., they had a lower rate of dental arch reduction. Thus, surgical time may interfere with the bone growth rate of the maxilla. Some studies [25,26] demonstrate a reduction of the dental arch after lip repair (linear measurements, C-C′ and I-CC′), but there are no studies comparing this rate in different surgical times for lip repair.

Analyzing the groups at Time 1, Group 1 showed a lower mean in some linear measurements (I-C′; I-C′ and C-C′) and the sum of the segment areas, which was expected, since the participants in Group 1 were evaluated at 3 months of life, compared to 6 months for Group 2 and between 8 and 10 months for Group 3, thus having a bone growth. When analyzed at Time 2, Groups 2 and 3 showed some larger linear measurements (I-C′) and the sum of the segment areas compared to Group 1. This indicates that there was more significant growth in the posterior maxilla and in the total area, which may suggest that lip repair performed at later ages can benefit from bone growth and maxillary development. The study by Hoffmannova et al. (2018) [27] indicates that early neonatal lip repair (average 3 days after birth) did not cause a reduction in length or width dimensions during the first year of life. These data suggest that a reconstructed lip has a natural formative effect on the actively growing anterior parts of the upper dental arch segments, which causes narrowing of the alveolar gap. However, in the study of Kotova et al. (2019) [28], comparing primary surgeries at 6 months, 3 months, and the neonatal period (average 3 days after birth), the authors found significantly smaller measurements in the group operated on in the neonatal period and 3 months, reinforcing the present results.

Analyzing the growth rate, Group 1 showed a higher rate in some measurements (I-C, C-C′, I-T′, I-T′, and I-TT′) compared to Groups 2 and 3. However, this growth rate was expected to be higher due to the period of analysis of the models. Time 1 for Group 1 was at 3 months, when the children were younger; thus, when compared from 5 years onward, it is expected that they would have a higher growth rate compared to the other groups, since Time 1 for Group 2 was at 5 and 6 months, and it was from 8 to 11 months for Group 3.

The Atack index is the gold standard for assessing the intermaxillary relationship of individuals with unilateral cleft lip and palate at 5 years [7]. Occlusal analysis via the Atack index is performed at age 5 years since this is the age when children present complete deciduous dentition. This index is measured on a scale ranging from 1 to 5 in which the higher the index, the worse the profile, ranging from regular occlusion to anterior and/or posterior crossbite [29]. The study of dental models plays a relevant therapeutic role in treating individuals with oral clefts since it points out dimensional changes and enables the use of indices regarding treatment [29]. In this study, we found a higher percentage (44%) of participants with index 4 (negative horizontal overjet, with buccal or normal inclination of the upper incisors, tendency to open bite on the cleft side, and tendency to unilateral or bilateral posterior crossbite) in Group 1. Conversely, in Groups 2 and 3, there was a highest percentage of patients with index 3 (anterior edge-to-edge relationship, buccal inclination of the upper incisors or horizontal overbite with palatally inclined incisors, and tendency to open bite on the cleft side). According to the index of Atack et al. (1997) [7], patients with indices 1 and 2 generally require simple orthodontic treatment for correction of the malocclusion. With index 3, orthodontic treatment is complex, while with indexes 4 and 5, patients have occlusal problems that are solved through the association of orthodontic treatment and orthognathic surgery [21,30].

The limitation of this study was the surgical time. This study highlights the benefits of later lip repair for maxillary growth. However, it is crucial to consider the individual holistically. Factors like nutrition, such as the transition to sucking and solid food intake around six months, and the psychological impact on parents when opting for delayed lip repair should be carefully analyzed. This study presented a trend towards occlusal indices with better results for maxillary growth and development for participants who underwent surgery later in life; however, confirmation of the present results is necessary since the hospital’s multidisciplinary team is constantly seeking to achieve esthetic and functional rehabilitation ever closer to the ideal, aiming at a better quality of life for patients [31,32,33].

Thus, according to the sample studied and the period of follow-up, the best results were found in children with cleft lip and palate operated on later. Future studies must take into account other parameters such as surgical techniques and the need for secondary plastic surgery.

## Figures and Tables

**Table 1:** Intragroup analysis of linear measurements (mm) and area (mm^2^) (paired *t*-test and Wilcoxon test).

	Group 1			Group 2			Group 3		
Measurements	Time 1 Mean/SD (Median/IR)	Time 2 Mean/SD (Median/IR)	*p*-Value	Time 1 Mean/SD (Median/IR)	Time 2 Mean/SD (Median/IR)	*p*-Value	Time 1 Mean/SD (Median/IR	Time 2 Mean/SD (Median/IR)	*p*-Value
I-C′	20.34/4.63	12.46/3.35	<0.0001 *	23.00/4.43	12.67/2.23	<0.0001 *	20.65/5.46	14.06/2.22	<0.0001 *
I-C	13.12/1.61	16.05/1.79	<0.0001 *	14.35/1.99	15.12/1.57	0.0056 *	14.37/3.09	15.77/2.71	0.0958
C-C′	29.10/4.12	25.66/3.62	<0.0001 *	32.46/3.72	25.90/2.99	<0.0001 *	30.24/4.95	27.10/3.01	0.0007 *
I-CC′	7.80/1.63	5.77/1.77	<0.0001 *	8.69/1.71	4.72/1.50	<0.0001 *	8.22/2.90	5.80/1.95	0.0009 *
I-T′	35.96/2.79	38.86/3.90	<0.0001 *	36.69/2.99	37.82/3.81	0.0084 *	27.02/2.78	39.25/3.34	0.0083 *
I-T	31.35/3.74	40.30/3.73	<0.0001 *	(31.05/4.29)	(37.38/5.04)	<0.0001 *†	(33.45/3.57)	(37.54/3.48)	<0.0001 *†
T-T′	33.27/3.65	46.72/4.48	<0.0001 *	32.11/3.34	41.86/3.47	<0.0001 *	31.97/5.07	42.86/3.36	<0.0001 *
I-TT′	28.74/3.31	32.43/3.71	<0.0001 *	28.99/2.97	30.92/3.58	0.0005 *	30.39/3.06	32.12/3.67	0.0732
Sum of segment areas	1044.07/182.38	923.12/205.02	0.0003 *	1161.21/293.56	1066.95/280.03	0.0466 *	(1269.14/273.26)	(1118.05/184.38)	0.0170 *†

SD, standard deviation; IR, interquartile range; †, Wilcoxon test. * Statistically significant difference.

**Table 2:** Intergroup analysis (Group 1 × Group 2 × Group 3) of linear dimensions (mm) and area (mm^2^) at Time 1 (ANOVA followed by Tukey’s test; Kruskal–Wallis test followed by Dunn’s test).

Measurements	Group 1 Mean/SD (Median/IR)	Group 2 Mean/SD (Median/IR)	Group 3 Mean/SD (Median/IR)	*p*-Value
I-C′	20.34/4.63 ^A^	23.00/4.43 ^B^	20.65/5.46 ^AB^	0.0140 *
I-C	13.12/1.61 ^A^	14.35/1.99 ^B^	14.37/3.09 ^B^	0.0082 *
C-C′	29.10/4.12 ^A^	32.46/3.72 ^B^	30.24/4.95 ^AB^	0.0004 *
I-CC′	7.80/1.63	8.69/1.71	8.22/2.90	0.0890
I-T′	35.33/4.05	35.82/3.95	36.80/2.78	0.2798
I-T	(31.27/5.71)	(31.05/4.29)	(33.45/3.57)	0.0585 §
T-T′	33.27/3.65	32.11/3.34	31.97/5.07	0.2323
I-TT′	28.74/3.31	28.99/2.97	30.39/3.06	0.856
P-P′	(9.89/4.88)	(7.80/6.46)	(9.08/5.71)	0.1888 §
U-U′	12.95/3.48	12.01/3.46	13.38/3.73	0.2688
Cleft Area	323.62/83.54 ^AB^	288.48/88.52 ^A^	339.23/95.29 ^B^	0.0346 *
Sum of segment areas	1045.87/147.28 ^A^	1157.91/211.26 ^B^	1252.32/183.74 ^B^	<0.0001 *

SD, standard deviation; IR, interquartile range; §, Kruskal–Wallis test. * Different capital letters in the row indicate statistically significant difference.

**Table 3:** Intergroup analysis (Group 1 × Group 2 × Group 3) of linear dimensions (mm) and area (mm^2^) at Time 2 (ANOVA followed by Tukey’s test and Kruskal–Wallis test followed by Dunn’s test).

Measurements	Group 1 Mean/SD (Median/IR)	Group 2 Mean/SD (Median/IR)	Group 3 Mean/SD (Median/IR)	*p*-Value
I-C′	12.46/3.35 ^A^	12.67/2.23 ^AB^	14.06/2.22 ^B^	0.0461 *
I-C	16.05/1.79	15.12/1.57	15.77/2.71	0.0563
C-C′	25.66/3.62	25.90/2.99	27.10/3.01	0.1736
I-CC′	5.77/1.77 ^A^	4.72/1.50 ^B^	5.80/1.95 ^A^	0.0039 *
I-T′	39.86/3.90 ^A^	37.82/3.81 ^B^	39.25/3.34 ^AB^	0.0251 *
I-T	(40.69/4.21) ^A^	(37.38/5.04) ^B^	(37.54/3.48) ^B^	0.0030 *§
T-T′	46.72/4.48 ^A^	43.86/3.47 ^B^	42.86/3.36 ^B^	<0.0001 *
I-TT′	32.43/3.71	30.92/3.58	32.12/3.67	0.1065
Sum of segment areas	(923.12/205.02) ^A^	(1066.95/280.03) ^B^	(1118.8/184.38) ^B^	<0.0001 *§

SD, standard deviation; IR, interquartile range; §, Kruskal–Wallis test. * Different capital letters in the row indicate statistically significant difference.

**Table 4:** Intergroup analysis (Group 1 × Group 2 × Group 3) of the percentage (%) growth rates of linear dimensions (mm) and area (mm^2^) (ANOVA followed by Tukey’s test and Kruskal–Wallis test followed by Dunn’s yest).

Measurements	Group 1 Mean/SD (Median/IR)	Group 2 Mean/SD (Median/IR)	Group 3 Mean/SD (Median/IR)	*p*-Value
I-C′	(−59.05/70.56) ^A^	(−45.80/15.34) ^A^	(−33.05/17.85) ^B^	0.0003 *§
I-C	(23.71/22.77) ^A^	(6.23/15.31) ^B^	(16.32/41.41) ^AB^	0.0001 *§
C-C′	(−11.31/19.88) ^A^	(−20.21/12.28) ^B^	(−11.64/12.24) ^A^	0.0002 *§
I-CC′	(−28.44/35.13) ^A^	(−47.10/19.26) ^B^	(−23.59/50.44) ^A^	0.0001 *§
I-T′	11.40/13.22	5.49/13.46	6.50/11.32	0.0617
I-T	(30.41/17.16) ^A^	(21.93/14.86) ^B^	(13.21/12.34) ^B^	<0.0001 *§
T-T′	41.47/15.81	38.11/18.23	36.46/18.81	0.4349
I-TT′	(14.14/16.52) ^A^	(4.20/16.33) ^AB^	(0.41/17.47) ^B^	0.0071 *§
Sum of segment areas	−8.33/17.20	−3.74/23.07	−7.38/14.98	0.4741

SD, standard deviation; IR, interquartile range; §, Kruskal–Wallis test. * Different capital letters in the row indicate statistically significant difference.

**Table 5:** Intergroup analysis (Group 1 × Group 2 × Group 3) of the Atack index (Kruskal–Wallis test followed by Dunn’s test).

Atack Index	Group 1, N (%)	Group 2, N (%)	Group 3, N (%)	*p*-Value
1	0 (0)	4 (8)	4 (15.4)	
2	3 (6)	11 (22)	8 (30.8)	
3	21 (42)	24 (48)	11 (42.4)	
4	22 (44)	10 (20)	1 (3.8)	
5	4 (8)	1 (2)	2 (7.6)	
Total participants	50 (100) ^A^	50 (100) ^B^	50 (100) ^B^	<0.0001 *

Different capital letters in the row indicate a statistically significant difference. * Statistically significant difference. N, number of participants; (%), percentage regarding N.

## Data Availability

Data are contained within the article.
